# Chronic d-ribose and d-mannose overload induce depressive/anxiety-like behavior and spatial memory impairment in mice

**DOI:** 10.1038/s41398-020-01126-4

**Published:** 2021-02-02

**Authors:** Ke Xu, Mingyang Wang, Wei Zhou, Juncai Pu, Haiyang Wang, Peng Xie

**Affiliations:** 1 Department of Neurology, Yongchuan Hospital of Chongqing Medical University, Chongqing, China; 2 NHC Key Laboratory of Diagnosis and Treatment on Brain Functional Diseases, The First Affiliated Hospital of Chongqing Medical University, Chongqing, China; 3 Institute of Neuroscience and Collaborative Innovation Center for Brain Science, Chongqing Medical University, Chongqing, China; 4 College of Biomedical Engineering, Chongqing Medical University, Chongqing, China; 5 Department of Neurology, The First Affiliated Hospital of Chongqing Medical University, Chongqing, China

**Keywords:** Neuroscience, Diseases

## Abstract

The effects of different forms of monosaccharides on the brain remain unclear, though neuropsychiatric disorders undergo changes in glucose metabolism. This study assessed cell viability responses to five commonly consumed monosaccharides—D-ribose (RIB), D-glucose, D-mannose (MAN), D-xylose and L-arabinose—in cultured neuro-2a cells. Markedly decreased cell viability was observed in cells treated with RIB and MAN. We then showed that high-dose administration of RIB induced depressive- and anxiety-like behavior as well as spatial memory impairment in mice, while high-dose administration of MAN induced anxiety-like behavior and spatial memory impairment only. Moreover, significant pathological changes were observed in the hippocampus of high-dose RIB-treated mice by hematoxylin-eosin staining. Association analysis of the metabolome and transcriptome suggested that the anxiety-like behavior and spatial memory impairment induced by RIB and MAN may be attributed to the changes in four metabolites and 81 genes in the hippocampus, which is involved in amino acid metabolism and serotonin transport. In addition, combined with previous genome-wide association studies on depression, a correlation was found between the levels of Tnni3k and Tbx1 in the hippocampus and RIB induced depressive-like behavior. Finally, metabolite–gene network, qRT-PCR and western blot analysis showed that the insulin-POMC-MEK-TCF7L2 and MAPK-CREB-GRIN2A-CaMKII signaling pathways were respectively associated with RIB and MAN induced depressive/anxiety-like behavior and spatial memory impairment. Our findings clarified our understanding of the biological mechanisms underlying RIB and MAN induced depressive/anxiety-like behavior and spatial memory impairment in mice and highlighted the deleterious effects of high-dose RIB and MAN as long-term energy sources.

## Introduction

With the improvement in global living standards, and apart from genetic factors, excess sugar consumption has been proposed as a potential risk factor for metabolic diseases^1^. Most people rely on naturally occurring fructose as an added sugar, which has prompted popular recommendations to limit the intake of fructose and added sugar sucrose, which is a major source of fructose^2^. However, between 2005 and 2010, average sugar consumption in developed countries was well over 10% among all age and sex groups^3^. d-glucose (Glc) is the most widely distributed monosaccharide in nature. Glc ingestion induces rapid changes in plasma glucose levels, insulin, and gut-derived hormones. The mammalian brain depends on Glc as its main source of energy^4^. As neuropsychiatric disorders are commonly associated with Glc metabolism dysregulation^5^, we are interested in the effects of other sugars on the brain, especially the hexoses and pentoses, two types of monosaccharides that can pass through the blood–brain barrier and enter the brain^6^.

Recently, d-ribose (RIB), d-mannose (MAN), d-xylose (XYL), and l-arabinose (ARA), five hexoses or pentoses, have been increasingly used as food additives or nutritional supplements. RIB is a naturally occurring pentose monosaccharide present in all living cells. Supplementation of RIB in habitual daily diets may be beneficial to maintain the necessary levels of ATP during high-intensity exercise^7^. MAN is a C-2 epimer of glucose that occurs naturally in many plants and fruits, and it has been used as a non-antibiotic treatment for bacterial urinary tract infections^8^. It also plays a central role in energy generation, storage, and cell regulation^9^. Furthermore, XYL is a natural pentose sugar that is abundant in plants, and it has been widely used as a sweetener. It has been reported to reduce postprandial Glc and to regulate serum insulin^10^. ARA is a naturally occurring plant pentose that has gained considerable attention as a functional food for intestinal health^11^. Noticeably, it has been reported that the average urine RIB level of Alzheimer’s disease (AD) patients (96.91 ± 17.36 µmol/L) was higher than that of cognitively normal participants (55.29 ± 7.08 µmol/L)^12^. Our recent non-targeted metabolomics analysis also found abnormally high concentrations of RIB in the hippocampus of rats with depression^13^. In addition, the MAN plasma level was significantly higher in obese subjects, which was associated with insulin resistance and secretion^14^. Although several studies have reported that RIB causes cognitive impairment^15,16^, the physiopathological mechanisms are still poorly understood.

The hippocampus is a critical brain region of the limbic system and it has a vital role in memory formation and mood regulation^17^. Both postmortem and high-resolution magnetic resonance imaging volumetric studies have consistently revealed a smaller hippocampus in patients with depressive or anxiety disorders^18^. Furthermore, the integration of metabolomics and transcriptomics has been shown to be an innovative way of determining phenotype-related gene functions and metabolic pathways based on a series of gene actions and their final products, metabolites^19^. To our knowledge, no study has been carried out to examine the association between MAN and neuropsychiatric disorders, or the use of metabolomics and transcriptomics to elucidate the underlying mechanisms of RIB and MAN induced neurological impairment in the mouse hippocampus.

In this study, we used CCK8 assay to investigate cell viability to assess responses to different concentrations of RIB, Glc, MAN, XYL, and ARA in cultured neuro-2a (N2a) cells. We also examined the effects of low- and high-dose RIB and MAN on depressive- and anxiety-like behavior, as well as spatial learning and memory ability. The studies also assessed the concentrations of RIB and MAN in hippocampus, prefrontal cortex, cerebral cortex, and hypothalamus after RIB and MAN injections. Furthermore, an integration of widely targeted metabolomics and transcriptomics in the hippocampus was performed to investigate the underlying molecular pathogenic mechanisms. The signaling pathways affected by RIB and MAN were validated using qRT-PCR and western blot analysis in vivo.

## Materials and methods

### Cell culture and cell viability assay

The mouse neuroblastoma cell line N2a culture and cell viability assay were performed as previously described^20^. See Supplementary Information for further details.

### Animals and treatment

**Fig. 1:**
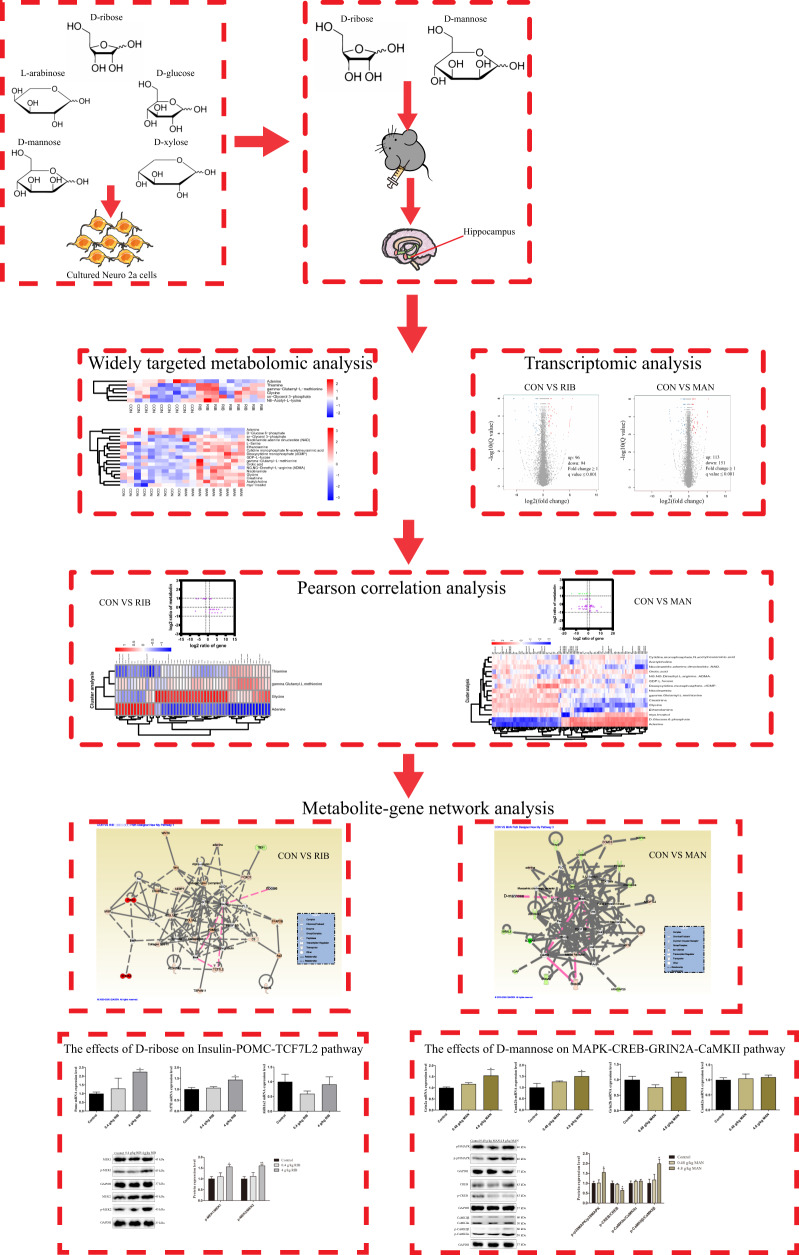
Experimental flow chart. The effects of D-ribose (RIB), L-arabinose (ARA), D-glucose (Glc), D-mannose (MAN) and D-xylose (XYL) on cell viability were evaluated in cultured neuro-2a (N2a) cells. Then, two of monosacccharides (RIB and MAN) were selected for intraperitoneal injection in mice, and the effects of these monosaccharides on depressive- and anxiety-like behavior, as well as spatial learning and memory ability were examined. In addition, the integration of widely targeted metabolomics and transcriptomics in the hippocampus were performed. Based on metabolite-gene network analysis, qRT-PCR and western blot analysis were used to verify the effects of RIB on the insulin-POMC-MEK-TCF7L2 pathway and the effects of MAN on the MAPK-CREB-GRIN2A-CaMKII pathway.

Male C57BL/6J mice (8–10 weeks) were maintained in approved animal facilities under controlled conditions (12 h light/dark cycle, 21–25 °C). After acclimatization, mice were randomly divided into five groups and, for 4 weeks, received either daily intraperitoneal (i.p.) injections of low-dose RIB (0.4 g/kg; *n* = 15) or high-dose RIB (4 g/kg; *n* = 22), low-dose MAN (0.48 g/kg; *n* = 14), or high-dose MAN (4.8 g/kg; *n* = 21) or 0.9% saline (control; *n* = 22). The i.p. application mode, dose, and time were selected from published studies^15,21^. After behavioral testing, mice were killed and their brains were immediately dissected out. Three randomly selected samples per group were fixed in 4% paraformaldehyde for histopathological examination. From the remaining samples, the hippocampus, prefrontal cortex, cerebral cortex, and hypothalamus were removed and frozen in liquid nitrogen, then stored at −80 °C until use. From the controls, 4 g/kg RIB and 4.8 g/kg MAN groups, we randomly selected nine hippocampus samples per group for widely targeted metabolomics and four hippocampus samples per group for transcriptomics. The samples for transcriptomics were from the same hippocampi used for widely targeted metabolomics. Based on past experience of previous studies, an appropriate number of samples from controls, 0.4 and 4 g/kg RIB, and 0.48 and 4.8 g/kg MAN groups were randomly selected for the molecular studies. The overall experimental process is presented in Fig. 1.

All animal experiments were approved by the Animal Care Committee of Chongqing Medical University and were conducted by adhering to the U.K. Animals (Scientific Procedures) Act, 1986, and ARRIVE (Animal Research: Reporting of In Vivo Experiments) guidelines.

### Food consumption test and sucrose preference test

Mouse food consumption and sucrose preference were monitored and recorded once per week throughout the experiment, consistent with our previous study^17^.

### Behavioral tests

After daily injection of either of the two concentrations of (i.e., RIB or MAN) for four weeks, mice were subjected to a series of behavioral tests (i.e., open-field test, elevated plus-maze, tail suspension test, and the Morris water maze) in a blinded manner by two experimenters. All behavioral tests were performed daily from 9:00 am to 4:00 pm with minimal stress. The interval was 24 h between tests to avoid the effect of the tail suspension test, and the interval between the Morris water maze and tail suspension test was 2 days. The detailed methods of these tests are shown in the Supplementary Information section.

### Ultra-performance liquid chromatography-mass spectrometry (UPLC-MS) analysis

This analysis was used to detect the concentration of RIB in the hippocampus, prefrontal cortex, cerebral cortex, and hypothalamus after RIB injection. Analysis was conducted using an Acquity UPLC I-Class (Waters Corp. Milford, MA) coupled to a Waters Xevo G2-S QTof mass spectrometry system operating in negative ion mode as previously described with minor modifications^22^. See Supplementary Information for further details.

### Hematoxylin–eosin (HE) staining

Similar to the previous description^23^, mouse brain was paraffin-embedded, sliced, dewaxed, and then stained using a HE detection kit (Servicebio, China). Finally, histopathological results of the CA1, CA2, CA3, and dentate gyrus were photographed (Nikon, Japan) and analyzed.

### Widely targeted metabolomics analysis

This analysis was performed at the Biotree Company (Shanghai, China), as previously described^24^. The metabolites with variable importance in the projection (VIP) > 1.0 and *P* < 0.05 (Student’s *t* test) were considered as significantly differentially expressed metabolites (DEMs). See Supplementary Information for further details.

### Enzyme-linked immunosorbent assay (ELISA)

This assay was used to detect the concentrations of MAN in the hippocampus, prefrontal cortex, cerebral cortex, and hypothalamus after MAN injection by utilizing an ELISA kit (Jonln, China). Moreover, to assess the reliability of the metabonomic analysis results, the concentrations of glycine and thiamine in the hippocampus of RIB-treated mice, and the concentrations of glycine, acetylcholine, creatinine, and nicotinamide in the hippocampus of MAN-treated mice were detected using ELISA kits (Jonln) according to the manufacturer’s instructions.

### RNA-sequencing analysis

The cDNA library construction and sequencing were performed by the Beijing Genomics Institute using BGISEQ-500 platform (Shenzhen, China)^19^. The raw data in this study can be available in NCBI SRA database with accession number of PRJNA639903. The threshold for significantly differentially expressed genes (DEGs) was set as the adjusted *P* value (*Q* value) ≤ 0.001 and |log2(fold change)| ≥ 1. See Supplementary Information for further details.

### Integrative analysis of metabolome and transcriptome

The Pearson method was used to integrate and analyze the correlation coefficients for the metabolome and transcriptome data from the same sample set. Correlation coefficients (*r*) were calculated from log2(fold change) of each DEM and log2(fold change) of each DEG, and correlations with |*r*| > 0.8 and *P* < 0.05 were selected for further analysis. Gene Ontology (GO) and Kyoto encyclopedia of genes and genomes (KEGG; http://www.genome.jp/kegg/) analysis were used to identify the potential functions of these selected DEGs and *P* value < 0.05 was set as the cutoff criterion. In addition, the protein–protein interaction (PPI) network (score > 500) and co-expression network (Pearson correlation coefficient |*r*| > 0.8) were used to construct an interaction network of these DEGs, and Cytoscape (http://cytoscape.org/) was used to mine the function modules^25^. Furthermore, based on Fischer’s exact test, Ingenuity Pathways Analysis (IPA; www.ingenuity.com) was used to analyze the integrated DEMs and DEGs to explore the significantly altered canonical pathways and metabolite–gene interaction networks^26^.

### qRT-PCR

Consistent with our previous study^17^, reverse transcription was performed using the PrimeScript™ RT Master Mix Kit (Takara, Japan), and the qRT-PCR was performed using the SYBR Green detection system (Roche, Germany). Glyceraldehyde-3-phosphate dehydrogenase was used as an internal control. The primer sequences are shown in Supplementary Table S1.

### Western blotting

For this analysis, the detailed process has been described previously^17^. Whole-cell lysis was performed to detect the expression levels of MAP kinase 1 (MEK1), phosphorylated MEK1, MEK2, phosphorylated MEK2, p38 mitogen-activated protein kinase (p38MAPK), phosphorylated p38MAPK, cAMP-responsive element binding protein (CREB), phosphorylated CREB, calcium/calmodulin-dependent protein kinase II α/β (CaMKIIα/β) and phosphorylated CaMKIIα/β. See Supplementary Information for further details.

### Statistical analysis

Data are expressed as mean ± S.E.M. An assessment of the normality of data was carried out using the Shapiro–Wilk test. Excepted as specially provided, data were analyzed by carrying out a one-way analysis of variance (ANOVA) or repeated measures two-way ANOVA followed by post hoc using GraphPad Prism 7 (San Diego, USA) software. The statistical significance was defined as *P* < 0.05.

## Results

### RIB and MAN significantly inhibited cell growth in N2a cells

**Fig. 2:**
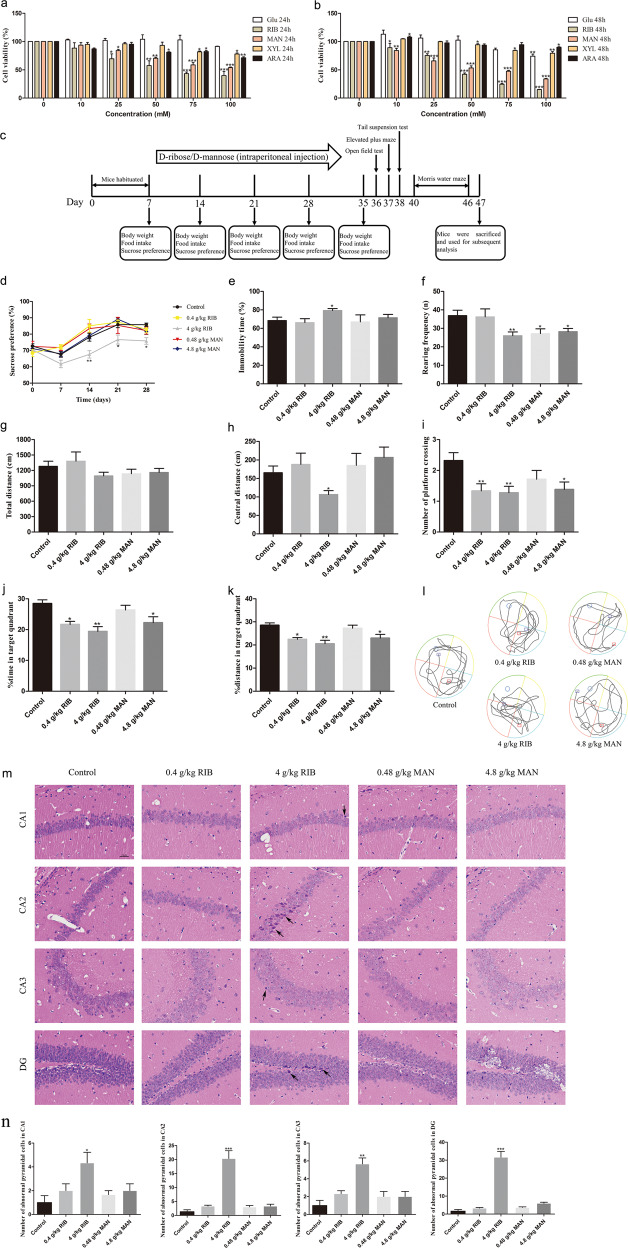
Effects of five monosaccharides on N2a cell viability and two doses of d-ribose (RIB) or d-mannose (MAN) on mouse depressive- and anxiety-like behavior, and spatial learning and memory. **a**, **b** N2a cells were incubated with different concentrations of d-glucose (Glc), RIB, MAN, d-xylose (XYL) or L-arabinose (ARA) for 24 h **a** or 48 h **b**, as indicated, and cell viability was measured using the CCK8 assay (*n* = 3 each group). **c** Timeline of the RIB and MAN treatments and behavioral tests in mice (0.4 g/kg RIB group: *n* = 15; 4 g/kg RIB group: *n* = 22; 0.48 g/kg MAN group: *n* = 14; 4.8 g/kg group: *n* = 21; control group: *n* = 22). **d** The sucrose preference test was performed weekly for 4 weeks. **e** The percentage of immobility time in the tail suspension test was recorded. **f**–**h** In the open-field test, the number of rearing **f**, and the total (**g**) and central (**h**) distance were recorded. **i**–**l** In the Morris water maze, the number of platform crossings (**i**), the percentage of time (**j**) and distance (**k**) spent in the target quadrant in the probe trial, as well as the typical trajectories (**l**) are shown. The red and blue squares, respectively, represent the starting and ending positions of the mice. **m** Representative hematoxylin–eosin staining image, which illustrates histopathological changes in the CA1, CA2, CA3, and dentate gyrus (DG) of the hippocampus. scale bar = 100 µm. Black arrows indicate significant histopathological changes. **n** Statistical analysis results of HE staining (*n* = 3 each group). Data represent mean ± S.E.M. *P < 0.05, **P < 0.01, ***P < 0.001 versus the control group.

To evaluate the effects of the five monosaccharides on N2a cell viability, we conducted the CCK8 assay. RIB and MAN, in contrast to the other monosaccharides, much more significantly reduced cell viability in a time- and dose-dependent manner (*P* < 0.05; Fig. 2a, b). Therefore, we selected RIB and MAN for the subsequent experiments to further investigate their potential detrimental effects on the brain.

### Effects of RIB and MAN on mouse body weight and food consumption

During the 4 weeks of RIB and MAN administration, mouse body weight and food consumption were measured weekly (Fig. 2c). There were no significant effects of RIB or MAN on body weight (*P* > 0.05; Supplementary Fig. S1a) or food consumption (*P* > 0.05; Supplementary Fig. S1b) during the experimental period.

### Effects of RIB and MAN on depressive- and anxiety-like behavior

The sucrose preference was significantly lower in the 4 g/kg RIB group than in the control group on days 14, 21, and 28 (*P* < 0.05; Fig. 2d). Furthermore, the percentage immobility time in the 4 g/kg RIB group was significantly higher than that in the control group (*P* < 0.05; Fig. 2e). However, significant changes were not observed in the low- or high-dose MAN groups. These results demonstrate that high-dose levels of RIB induced depressive-like behavior in mice.

In the open-field test, the number of rearing was significantly decreased in the 0.4 and 4 g/kg RIB groups and in the 4.8 g/kg MAN group compared with controls (*P* < 0.05; Fig. 2f). There were no differences in the total distance among the groups (*P* > 0.05; Fig. 2g), whereas the central distance in the 4 g/kg RIB group was significantly lower than that in the control group (*P* < 0.05; Fig. 2h). Moreover, we carried out the elevated plus-maze test, and the time spent in the open arms was measured. Compared with the control group, there were no significant differences in the time spent in the open arms in the low- or high-dose RIB or MAN groups (*P* > 0.05; Supplementary Fig. S1c).

### Effects of RIB and MAN on spatial learning and memory

To investigate the effects of RIB and MAN on spatial learning and memory, the Morris water maze was performed. During the 5 days of training trials, all mice improved their performance as indicated by increasingly shorter escape latencies over successive days. Compared with the control group, no difference was found in escape latency in the 0.4 and 4 g/kg RIB groups or the 0.48 and 4.8 g/kg MAN groups (*P* > 0.05; Supplementary Fig. S1d), suggesting that RIB and MAN did not significantly affect spatial learning ability.

In the probe trial, the 0.4 and 4 g/kg RIB groups and the 4.8 g/kg MAN group all displayed a significant impairment in spatial memory function, shown as decreased frequency of target platform crossings (*P* < 0.05; Fig. 2i) as well as lower percent time and distance traveled in the target quadrant, compared with the control group (*P* < 0.05; Fig. 2j, k). The typical trajectories of mice in the probe trial are shown in Fig. 2l.

### Levels of RIB and MAN in the brain after treatment

The UPLC-MS analysis (Supplementary Fig. S1e) indicated that the levels of RIB were significantly increased in the cerebral cortex, hippocampus, and hypothalamus after treatment (*P* < 0.05), whereas there was no significant difference in the prefrontal cortex (*P* > 0.05). Moreover, the ELISA result (Supplementary Fig. S1f) indicated that the levels of MAN were significantly increased in the cerebral cortex, hippocampus, and prefrontal cortex after treatment (*P* < 0.05), whereas there was no significant difference in the hypothalamus (*P* > 0.05). Compared with other brain regions, there was a higher concentration of RIB in the hippocampus and a higher concentration of MAN in the prefrontal cortex.

### Hippocampal histopathological analysis

The hippocampal sections were analyzed using HE staining methods (×400). We found obviously condensed and deeply-stained pyramidal cells in the hippocampus of mice in the 4 g/kg RIB group (*P* < 0.05), whereas there were no significant changes in the other groups (Fig. 2m, n).

### Metabonomic analysis of hippocampus from RIB- and MAN-treated mice

From UHPLC-MRM-MS/MS metabolomic profiling, 157 peaks were detected in this study (Supplementary Table S2), and 92 metabolites were left after relative standard deviation de-noising (Supplementary Table S3). Principal component analysis between RIB versus control (CON) groups and MAN versus CON groups are shown in Supplementary Fig. S2a. The resulting orthogonal projections to latent structures-discriminant analysis (OPLS-DA) score plot (Supplementary Fig. S2b) shows a clear separation between the RIB and CON groups (*R*^2^*X* = 0.381, *R*^2^*Y* = 0.774, *Q*^2^ = 0.182), as well as between the MAN and CON groups (*R*^2^*X* = 0.361, *R*^2^*Y* = 0.893, *Q*^2^ = 0.484). In addition, all samples were within the 95% confidence interval. The permutation tests also showed that the OPLS-DA model had good predictability and did not overfit (RIB versus CON, *R*^2^ = 0.79, *Q*^2^ = −0.51; MAN versus CON, *R*^2^ = 0.73, *Q*^2^ = −0.71; Supplementary Fig. S2c).

**Fig. 3:**
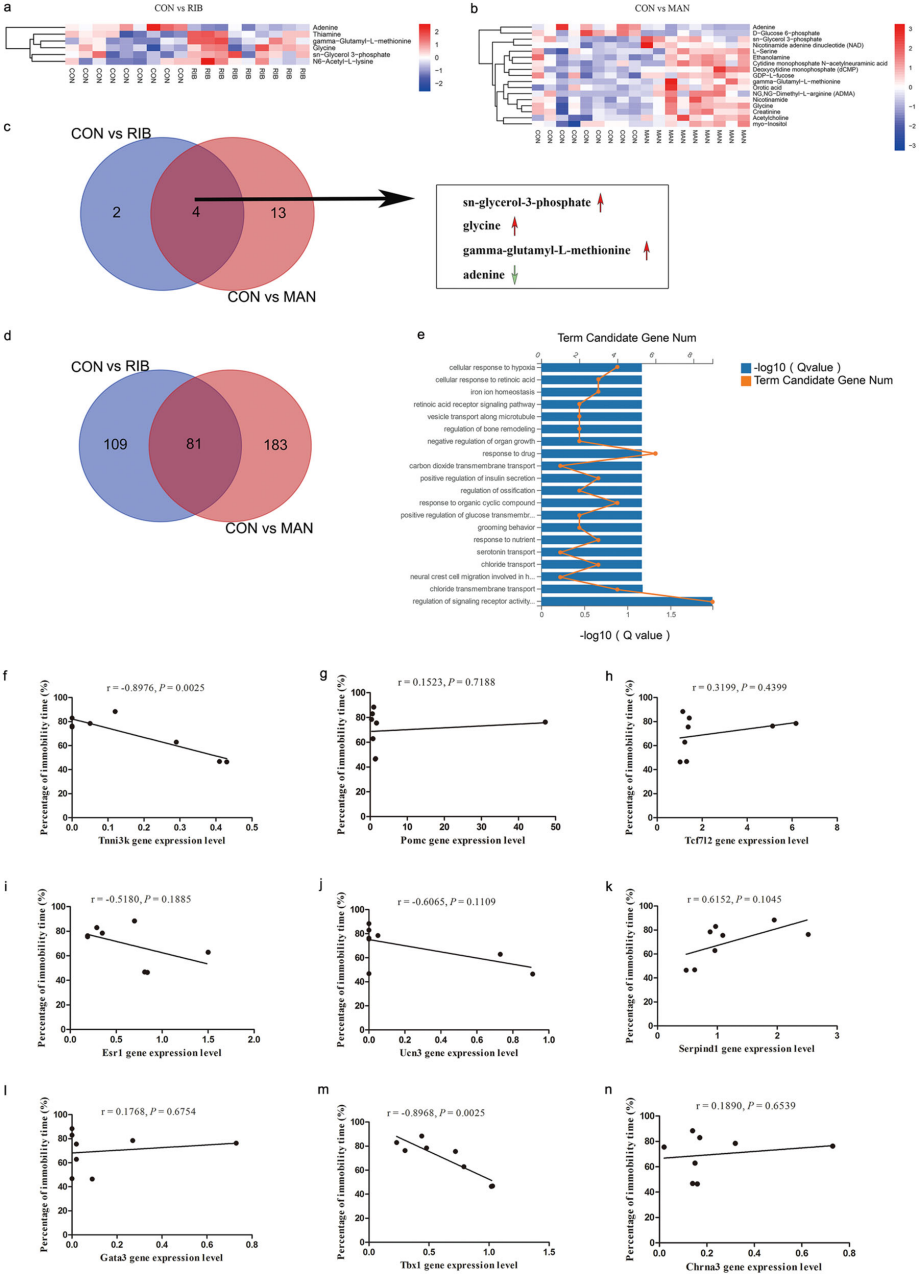
Widely targeted metabolomics and transcriptomics analysis of hippocampus from 4 g/kg d-ribose (RIB), 4.8 g/kg d-mannose (MAN) and control (CON) groups mice. **a**, **b** Heatmap of the six differentially expressed metabolites (DEMs) from the RIB versus CON **a** groups, and the 17 DEMs from the MAN versus CON **b** groups. The rows indicate the metabolites, and the columns indicate the samples of each group. **c** The Venn diagram indicates the three upregulated (sn-glycerol-3-phosphate, glycine and gamma-glutamyl-l-methionine) and one downregulated (adenine) metabolites in both the RIB and MAN groups. **d** The Venn diagram showing the 81 differentially expressed genes (DEGs) common to both the RIB and MAN groups. **e** Top 20 GO biological processes of the 81 genes altered in both the RIB and MAN groups (*P* < 0.05). **f**–**n** Correlations between the nine altered genes (Tnni3k (**f**), Pomc (**g**), Tcf7l2 (h), Esr1 (**i**), Ucn3 (**j**), Serpind1 (**k**), Gata3 (**l**), Tbx1 (**m**), and Chrna3 (**n**)) in the hippocampus and the percentage of immobility time of mice in the CON and RIB groups (*n* = 4 each group). *Chrna3* cholinergic receptor nicotinic alpha 3 subunit, *Esr1* estrogen receptor 1, *Gata3* GATA-binding protein 3, *Pomc* proopiomelanocortin, *Serpind1* serpin family D member 1, *Tbx1* T-box transcription factor 1, *Tcf7l2* transcription factor 7 like 2, *Tnni3k* TNNI3 interacting kinase, *Ucn3* urocortin 3.

Six DEMs were identified between the RIB and CON groups (five upregulated metabolites and one downregulated metabolite; Supplementary Table S4), and 17 DEMs were identified between the MAN and CON groups (15 upregulated metabolites and two downregulated metabolites; Supplementary Table S5). The ELISA results showed that two of the six DEMs in the RIB versus the CON group, and four of the 17 DEGs in the MAN versus the CON group selected for ELISA were consistent with the metabonomic analysis (Supplementary Fig. S2d–h), which verified that the metabonomic analysis was reliable. Heatmap visualization clearly shows these differential metabolites (Fig. 3a, b). Furthermore, we compared the DEMs for RIB versus CON and MAN versus the CON groups (Fig. 3c). This comparison revealed that three metabolites (sn-glycerol-3-phosphate, glycine and gamma-glutamyl-l-methionine) were upregulated, whereas adenine was downregulated in both the RIB and MAN groups.

### Transcriptomics analysis of hippocampus from RIB- and MAN-treated mice

We identified 190 DEGs in the RIB-treated mice compared with CON mice (Supplementary Table S6), and 264 DEGs in the MAN-treated mice compared with CON mice (Supplementary Table S7). The DEGs resulting from RIB and MAN treatment were visualized as volcano plots, which revealed 96 upregulated genes and 94 downregulated genes for RIB versus CON (Supplementary Fig. S2i), and 113 upregulated genes and 151 downregulated genes for MAN versus CON (Supplementary Fig. S2j). The results of the RT-qPCR assay showed that 15 of the 20 DEGs in the RIB versus CON group, and 14 of the 20 DEGs in the MAN versus CON group selected for RT-qPCR conformed to the RNA-Seq data (Supplementary Fig. S2k–n). These results indicated that the RNA-Seq was reliable. Top 20 GO analysis showed that the DEGs for RIB versus CON were enriched in biological processes such as “regulation of insulin secretion” (Supplementary Fig. S2o). Similarly, the DEGs for MAN versus CON could be mapped to biological processes such as “regulation ion and chloride transport” (Supplementary Fig. S2p).

Moreover, we compared the DEGs from the RIB versus CON as well as the MAN versus CON groups (Fig. 3d), which revealed 81 DEGs common to both the RIB and MAN groups (Supplementary Table S8). Top 20 GO analysis showed that these 81 DEGs were enriched in biological processes such as “regulation of signaling receptor activity” and “serotonin transport” (Fig. 3e). Given that only RIB induced depressive-like behavior in mice, we further investigated the possible mechanism. Recent genome-wide association studies^27–34^ on depression have implicated nine candidate genes (i.e., TNNI3 interacting kinase (Tnni3k), proopiomelanocortin (Pomc), transcription factor 7 like 2 (Tcf7l2), estrogen receptor 1, urocortin 3, serpin family D member 1, GATA-binding protein 3, T-box transcription factor 1 (Tbx1), and cholinergic receptor nicotinic alpha 3 subunit) among the 109 unique DEGs from the RIB versus CON groups. We analyzed the Pearson’s correlation coefficient between the percentage of immobility time, as determined by the tail suspension test, and the expression levels of these nine genes (Fig. 3f–n), which indicated that only Tnni3k and Tbx1 were significantly correlated with RIB induced depressive-like behavior (*P* < 0.05; Fig. 3f, m).

### Integrating metabolomics and transcriptomics data

Next, we performed correlation analysis using the DEMs and DEGs identified above to provide further insight into the molecular functional changes induced by the sugars. Using the Pearson method, we identified 51 DEGs that were highly correlated with four DEMs between the RIB and CON groups (Supplementary Table S9), as well as 109 DEGs that were highly correlated with 15 DEMs between the MAN and CON groups (Supplementary Table S10). The nine quadrant diagrams and heatmap of these metabolites and corresponding genes are shown in Supplementary Fig. S3a–d.

**Fig. 4:**
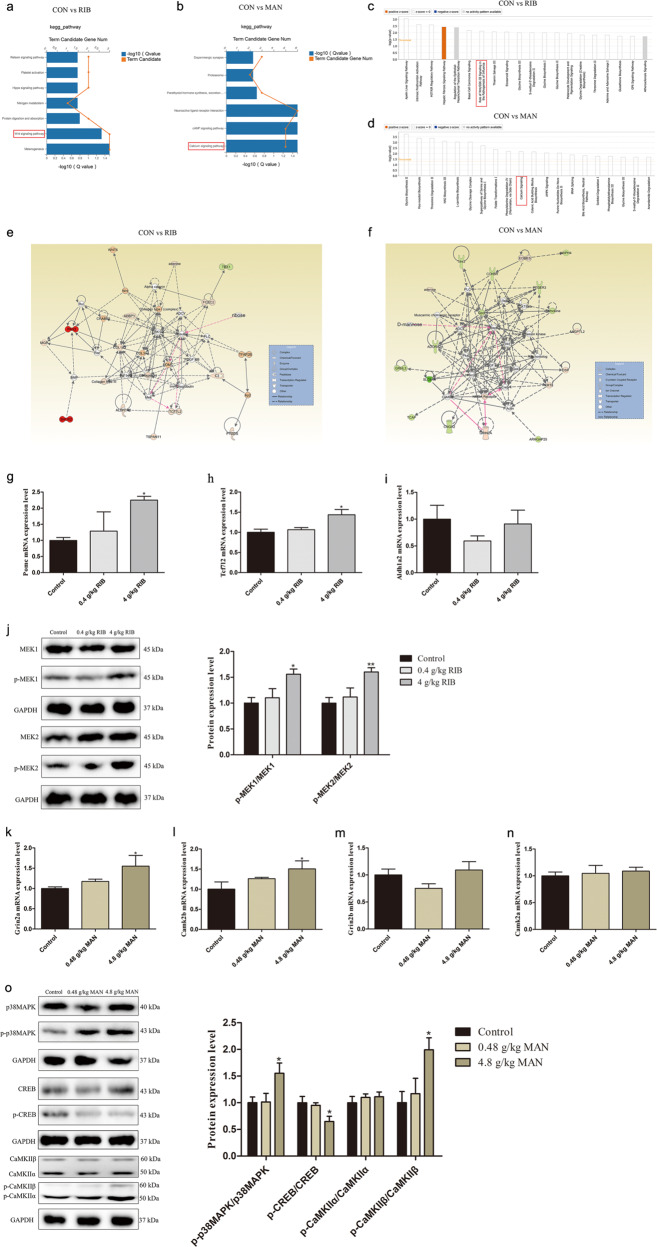
Integrated analysis of widely targeted metabolomics and transcriptomics data. **a**, **b** KEGG enrichment analysis of the selected 51 differentially expressed genes (DEGs) that were highly correlated with the differentially expressed metabolites (DEMs) from d-ribose (RIB) versus control (CON) groups (**a**; *P* < 0.05), and the selected 109 DEGs that were highly correlated with the DEMs from d-mannose (MAN) versus CON groups (**b**; *P* < 0.05). **c**, **d** Top 20 canonical pathways analysis of the selected DEGs with corresponding DEMs from RIB versus CON (**c**) and MAN versus CON (**d**) groups. **e** Top-ranked enriched metabolite–gene integrated network based on the selected DEGs with the corresponding DEMs from the RIB versus CON group comparison. Red lines indicate a possible insulin-POMC-MEK-TCF7L2 pathway in the underlying pathogenesis. **f** Top-ranked enriched metabolite–gene integrated network based on the selected DEGs with the corresponding DEMs from the MAN versus CON group comparison. Red lines indicate a possible MAPK-CREB-GRIN2A-CaMKII pathway in the underlying pathogenesis. **g**–**i** mRNA expression levels of Pomc (**g**), Tcf7l2 (**h**), and Aldh1a2 (**i**) in the control, 0.4 g/kg RIB and 4 g/kg RIB groups. **j** Phosphorylation levels of MEK1 and MEK2 in the control, 0.4 g/kg RIB and 4 g/kg RIB groups. **k**–**n** mRNA expression levels of Grin2a (**k**), Camk2b (**l**), Grin2b (**m**), and Camk2a (**n**) in the control, 0.48 g/kg MAN and 4.8 g/kg MAN groups. **o** Phosphorylation levels of p38MAPK, CREB, CaMKIIα, and CaMKIIβ in the control, 0.48 g/kg MAN and 4.8 g/kg MAN groups. *n* = 4 each group. Data represent mean ± S.E.M. * *P* < 0.05, **P < 0.01 versus the control group.

The results of the top 20 GO analysis for cellular components, molecular functions, and biological processes related to the RIB versus CON and the MAN versus CON groups are shown in Supplementary Fig. S4a–f. Notably, the GO terms indicated that the 51 DEGs for the RIB versus CON groups were enriched in regulation of transcription factors, especially TCF7L2. KEGG pathways analysis revealed that the 51 DEGs for the RIB versus CON groups were enriched in “Wnt signaling pathway,” and so on (Fig. 4a). The 109 DEGs for the MAN versus CON groups were enriched with respect to the “calcium signaling pathway,” and so on (Fig. 4b). Furthermore, the PPI and co-expression network analysis identified the possible relationship among these selected DEGs for the comparisons between the RIB versus CON groups (Supplementary Fig. S5a) and the MAN versus CON (Supplementary Fig. S5b) groups.

The selected DEGs and DEMs were submitted to IPA for integration canonical pathway analysis. Together with the GO, KEGG, co-expression, and PPI network results, the findings suggest that RIB is involved in the “Wnt signaling pathway” (Fig. 4c) and that MAN is involved in the “calcium signaling pathway” (Fig. 4d). Furthermore, based on the metabolite–gene integrated network analysis, the insulin-POMC-MEK-TCF7L2 pathway is probably implicated in the underlying pathogenesis of RIB (Fig. 4e), and the MAPK-CREB-glutamate ionotropic receptor NMDA type subunit 2A (GRIN2A)-CaMKII pathway is likely involved in the underlying pathogenesis of MAN (Fig. 4f).

### The insulin-POMC-MEK-TCF7L2 pathway levels in low- and high-dose RIB groups

Compared with the control group, the mRNA levels of Pomc and Tcf7l2 were both significantly increased in the 4 g/kg RIB group (*P* < 0.05; Fig. 4g, h), although there was no significant difference in the 0.4 g/kg RIB group. Given that PPI, co-expression network analysis, and IPA network analysis indicated that aldehyde dehydrogenase 1 family member a2 (Aldh1a2) was correlated with TCF7L2, we examined the mRNA levels of Aldh1a2 in the 0.4 and 4 g/kg RIB groups. There was no significant difference in Aldh1a2 mRNA levels between the groups (*P* > 0.05; Fig. 4i).

Moreover, western blot analysis confirmed that, compared with the control group, the phosphorylation levels of MEK1 and MEK2 were increased in the 4 g/kg RIB group (*P* < 0.05; Fig. 4j), although there was no significant difference in the 0.4 g/kg RIB group.

### The MAPK-CREB-GRIN2A-CaMKII pathway levels in low- and high-dose MAN groups

Compared with the control group, the mRNA levels of Grin2a and Camk2b were significantly increased in the 4.8 g/kg MAN group (*P* < 0.05; Fig. 4k, l), although there was no significant difference in the 0.48 g/kg MAN group. The mRNA levels of Grin2b and Camk2a had not shown any significant differences between the groups (*P* > 0.05; Fig. 4m, n).

The phosphorylation levels of p38MAPK, CREB, CaMKIIα, and CaMKIIβ were analyzed using western blotting (Fig. 4o). Compared with the control group, the phosphorylation levels of p38MAPK and CaMKIIβ were increased (*P* < 0.05), whereas the phosphorylation level of CREB was decreased in the 4.8 g/kg MAN group (*P* < 0.05), although there was no significant difference in the 0.48 g/kg MAN group (*P* > 0.05). In addition, there was no significant difference in the phosphorylation level of CaMKIIα between the groups (*P* > 0.05).

## Discussion

Perturbations of glucose metabolism have been linked to neuropsychiatric disorders^5^. However, the effects of other monosaccharides on the brain are still unknown. Several recent studies have reported that high levels of RIB induced cognitive impairment^15,20^, and that it is a potential biomarker for major depressive disorder^13^, whereas the possible underlying pathogenetic mechanisms remain poorly understood. Here, we showed that two commonly consumed monosaccharides, namely, RIB and MAN, significantly reduced N2a cell viability, which indicated that both of these sugars might have detrimental effects on the brain. We further evaluated the behavioral effects of RIB and MAN in mice and explored the underlying pathogenic mechanisms.

There were no significant differences in body weight or food intake in either the RIB or MAN groups. This finding is inconsistent with previous studies, which found that oral supplementation with RIB and MAN suppressed high-fat-diet-induced body weight gain in mice^35,36^. The discrepancies may not only be due to differences in the dosage of RIB and MAN and the administration regimen; the gut might also play a vital role. We speculated that i.p. administration of RIB and MAN would not effectively alter the gut microbiome and accelerate gut motility like oral administration. Further investigation was required. Moreover, we not only found that high-dose RIB induced depressive-like behavior, but also that the high-dose RIB and MAN groups exhibited anxiety-like behavior in the open-field test, although not in the elevated plus-maze. The contradictory results might be owing to differences between laboratories in the selection and analysis of behavioral parameters^37^. Furthermore, we only observed the effects of RIB and MAN on C57BL/6J mice: It should be emphasized that significant differences have been found between the three C57BL/6 sub-strains in behavioral tests, particularly in open-field test and the elevated plus-maze^38–41^. High-dose RIB and MAN significantly impaired spatial memory in the Morris water maze, but did not affect spatial learning. This finding is similar to that of a previous study^42^ in which inhibition of myelination impaired spatial memory without affecting spatial learning in adult mice. It remains to be determined whether the RIB and MAN induced spatial memory impairment involves inhibition of myelination or the promotion of myelin degeneration. We also confirmed that i.p. administration of RIB and MAN led to an increase in the concentrations of RIB and MAN in the mouse hippocampus, which verified the previous study’s findings that RIB and MAN can pass through the blood–brain barrier and enter the brain tissue by simple diffusion^6^. And the impact of other brain regions that influenced by RIB or MAN merits further investigation. In addition, HE staining showed that only high-dose RIB induced apoptosis of mice hippocampal neurons, which further confirmed that RIB has detrimental effects on the brain. The reason that mice are more sensitive to RIB than MAN under similar conditions can probably be ascribed to the most rapid glycation of RIB compared with others monosaccharides, and glycation plays an important role in the cytotoxicity of neural cells^43^.

The dose of RIB and MAN used in mice translates to 28 g/d and 280 g/d for a human body weight of 70 kg. The statement of the European Food Safety Authority^44^ reported that RIB was safe for the general population at intake levels up to 252 g/d. Previous results found that RIB doses of 336 g/d caused diarrhea in healthy subjects^45^. In patients with AD, the urine concentrations of RIB were found to be significantly increased as compared with cognitive normal controls. Increased RIB levels correlated with poor cognitive ability, especially in female AD patients^12^. These results suggest that RIB might be a new potential diagnostic biomarker for AD. What might be the reasons for the different effects of RIB on the female and male AD patients? We reasoned that estrogen might play an important role in modulating RIB function, which needs further in-depth investigation. In mice, gavage of RIB has been reported to induce AD-associated proteins like Aβ and Tau forming deposits, corresponding to memory loss^16^. Furthermore, in human subjects with obesity, MAN plasma levels and insulin resistance were shown to be associated^14^. All of these findings further suggest that the detrimental effects of high concentrations of RIB and MAN in the body cannot be ignored. Among the significantly altered metabolites and genes in the RIB and MAN groups, we found four DEMs (i.e., sn-glycerol-3-phosphate, glycine, gamma-glutamyl-l-methionine and adenine) and 81 DEGs in both the RIB and MAN groups. Sn-glycerol-3-phosphate is an important metabolite involved in the glycolysis pathway that affects signal transduction and brain development^46^. The combination of RIB and adenine promotes adenosine release, which acts as an important neuromodulator on hippocampal neuronal excitability and synaptic plasticity^47^. The other two metabolites were mainly categorized as amino acids. Perturbed amino-acid metabolism has been implicated in the development of psychiatric diseases and AD^26,48^. The 81 DEGs in both the RIB and MAN groups were involved in serotonin transport, which is also reported to be related to psychiatric diseases and AD^17,49^. Thus, the anxiety-like behavior and spatial memory impairment induced by both high-dose RIB and MAN may involve common mechanisms of amino-acid metabolism and serotonin system dysregulation in the hippocampus. Moreover, we found that the expression levels of Tnni3k and Tbx1 in the hippocampus were correlated with RIB induced depressive-like behavior. As RIB is an important component of ribonucleotides, we propose that the high doses of RIB induced depressive-like behavior may not only be associated with changes in the levels of Tnni3k and Tbx1, but may also be related to single-nucleotide polymorphism variation of these two genes.

The multi-functional enrichment analysis indicated that the effects of RIB were implicated in the Wnt signaling pathway and TCF7L2. It has been reported that the TCF7L2 is a key transcriptional effector of the Wnt signaling pathway^50^, which is linked to psychiatric diseases and AD^51,52^. Based on the metabolite–gene network analysis, RIB induced depressive- and anxiety-like behavior and spatial memory impairment might be mediated by the insulin-POMC-MEK-TCF7L2 pathway. Long-term oral administration of RIB increases serum insulin in mice^16^, and insulin regulates POMC neuronal plasticity to control glucose metabolism^53^. A meta-analysis of a genome-wide association study also revealed POMC and TCF7L2 that are associated with mood disorders^28^. Indeed, in this study, we found that Pomc and Tcf7l2 mRNA levels were both increased in the high-dose RIB group. In addition, high-dose RIB induced the activation of MEK1 and MEK2. Several studies have shown that activation of MEK1/2 is involved in depressive- and anxiety-like behavior and memory function in animal models^54–56^. Thus, the activation of MEK1/2 by RIB may also contribute to the depressive- and anxiety-like behavior and spatial memory impairment in mice.

Moreover, this study indicated that the effects of MAN are involved in the regulation of calcium signaling, which is correlated with neurodegenerative and neuropsychiatric disorders^57^. According to the metabolite–gene network, MAN induced anxiety-like behavior and spatial memory impairment, possibly via the MAPK-CREB-GRIN2A-CaMKII pathway. We confirmed that CaMKIIβ protein was activated in the high-dose MAN group. CaMKIIβ is believed to regulate neurotransmission and synaptic plasticity in response to calcium signaling produced by neuronal activity^58,59^. Furthermore, high-dose MAN increased Grin2a mRNA levels without impacting Grin2b. GRIN2A and GRIN2B are two important glutamate ionotropic receptor NMDA type subunits, which can affect synaptic plasticity by regulating binding to CaMKII^60^. Moreover, high-dose MAN increased the phosphorylation level of p38MAPK, but decreased the phosphorylation level of CREB. As p38MAPK activation is critical for NMDA receptor-dependent cognitive functions^61^, and NMDA receptor related-CREB pathway suppression might be correlated with hippocampal functional impairments^62^, the anxiety-like behavior and spatial memory impairment induced by MAN may also involve upregulation of GRIN2A in the hippocampus.

Here, we investigated the underlying pathogenesis using an integrated metabolomics and transcriptomics approach. Considering that RIB and MAN induced depressive/anxiety-like behavior and spatial memory impairment, which, respectively, correlated with the insulin-POMC-MEK-TCF7L2 and MAPK-CREB-GRIN2A-CaMKII pathways, the issue of whether inhibition of these pathways could circumvent the effects of RIB and MAN requires further investigation in future studies.

**Fig. 5:**
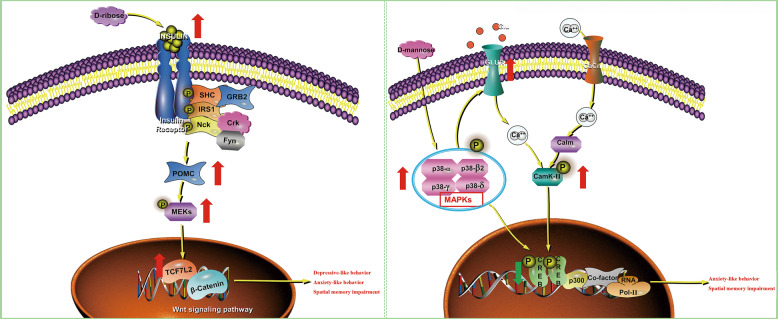
Brief summary of the research. The left panel shows that d-ribose induced depressive- and anxiety-like behavior and spatial memory impairment in mice by regulating the insulin-POMC-MEK-TCF7L2 pathway in the hippocampus. The right panel illustrates that d-mannose induced anxiety-like behavior and spatial memory impairment in mice by regulating the MAPK-CREB-GRIN2A-CaMKII pathway in the hippocampus. The red arrows indicate upregulated metabolites/genes and the green arrows indicate downregulated metabolites/genes. *Cacn* calcium channel, *Calm* calmodulin, *Crk* CT10 sarcoma oncogene cellular homolog, *Fyn* a Src family tyrosine-protein kinase, *GRB2* growth factor receptor-bound protein 2, *iGLUR* ionic glutamate receptor, *IRS1* recombinant insulin receptor substrate 1, *SHC* SH2-containing collagen-related proteins.

Overall (Fig. 5), our findings show that high-dose RIB induced depressive- and anxiety-like behavior and spatial memory impairment in mice, and highlight the involvement of the insulin-POMC-MEK-TCF7L2 pathway in the hippocampus. In contrast, high-dose MAN induced anxiety-like behavior and spatial memory impairment in mice, and the results suggest the involvement of the MAPK-CREB-GRIN2A-CaMKII pathway in the hippocampus. Even if further research is warranted to verify these findings, ample attention should be given to the detrimental effects of high-dose RIB and MAN.

## Supplementary information

Supplementary Information

Supplementary Materials and Methods

Supplementary Figure Legends

Supplementary Table S1

Supplementary Table S2

Supplementary Table S3

Supplementary Table S4

Supplementary Table S5

Supplementary Table S6

Supplementary Table S7

Supplementary Table S8

Supplementary Table S9

Supplementary Table S10

Supplementary Figure S1

Supplementary Figure S2

Supplementary Figure S3

Supplementary Figure S4

Supplementary Figure S5
