# Supplementary material for: Chronic d-ribose and d-mannose overload induce depressive/anxiety-like behavior and spatial memory impairment in mice
Source: Transl Psychiatry. 2021 Feb 2;11:90. doi: 10.1038/s41398-020-01126-4 (PMC7854712; doi:10.1038/s41398-020-01126-4)
Supplement: Supplementary file 1 — Supplementary Information [file 41398_2020_1126_MOESM1_ESM.docx]

**Supplementary information**

**Supplementary materials and methods**

**Supplementary figure legends**

**Supplementary Table S1**

**Supplementary Table S2**

**Supplementary Table S3**

**Supplementary Table S4**

**Supplementary Table S5**

**Supplementary Table S6**

**Supplementary Table S7**

**Supplementary Table S8**

**Supplementary Table S9**

**Supplementary Table S10**

**Supplementary Fig. S1**

**Supplementary Fig. S2**

**Supplementary Fig. S3**

**Supplementary Fig. S4**

**Supplementary Fig. S5**
