# Supplementary material for: Chronic d-ribose and d-mannose overload induce depressive/anxiety-like behavior and spatial memory impairment in mice
Source: Transl Psychiatry. 2021 Feb 2;11:90. doi: 10.1038/s41398-020-01126-4 (PMC7854712; doi:10.1038/s41398-020-01126-4)
Supplement: Supplementary file 2 — Supplementary Materials and Methods [file 41398_2020_1126_MOESM2_ESM.docx]

**Chronic D-ribose and D-mannose overload induce depressive/anxiety-like behavior and spatial memory impairment in mice**

***Supplemental Information***

**Supplementary Materials and Methods**

**Cell culture** **and cell viability assay**

Mouse neuroblastoma cell line N2a cells (Type Culture Collection of the Chinese Academy of Sciences, China) were cultured in Dulbecco’s modified Eagle’s medium (Hyclone, USA) supplemented with 10% fetal bovine serum (Gibco, USA), penicillin (100 IU/ml) and streptomycin (100 µg/ml) at 37 °C in a humidified 5% CO_2_ incubator.

Based on previous study^1^, N2a cells were seeded in 96-well plates and exposed to various concentrations (0, 10, 25, 50, 75, 100 mM) of D-glucose (Glc), D-ribose (RIB), D-mannose (MAN), D-xylose (XYL) or L-arabinose (ARA) (all purchased from Sigma-Aldrich, St. Louis, USA) for 24 and 48 h. Thereafter, 10 μL of cell counting kit 8 (CCK8; Dojindo, Japan) solution was added to each well. The absorbance at 450 nm was measured by using a Multiskan Go microplate reader (Thermo Fisher Scientific, Winooski, VT, USA).

**Sucrose preference test and food consumption test**

As previous description^2, 3^, anhedonia was determined by using the sucrose preference test. Before the first formal experiment, mice from each group were trained to choose between water and a 1% sucrose solution after initial habituation to two bottles of water for 24 h. For each sucrose preference and food consumption tests, after 12 h of food and water deprivation, mice were given enough food, water and 1% sucrose solution for 12 h. Mice food consumption was recorded and the sucrose preference was quantified according to the following formula: sucrose preferences (%) = sucrose consumption/(sucrose + water consumption) × 100%.

**Open-field test**

Mice were placed in an open-field apparatus (50 × 50 × 40 cm) for 5 min 30 s, with the first 30 s for adaptation. The locomotor activity was video-recorded using an automated tracking system (Noldus Ethovision XT11.5, Noldus Information Technology B.V., Netherlands), and the number of rearing, and the total and central distance traveled were measured.

**Elevated plus-maze**

This test was performed to evaluate anxiety-like behavior as previously described^4^. The apparatus is elevated to a height of 15 cm above the floor with four arms (30 × 6 cm) that is two open arms across from each other and perpendicular to two closed arms with a center platform (5 × 5 cm). Mice were placed in the central zone with head toward a closed arm, allowed to freely explore the maze for 5 min 30 s, with the first 30 s for adaptation. The total time spent in open arms was measured by Noldus video-tracking system.

**Tail suspension test**

Mice were suspended by the tails using adhesive tape for 6 min and the initial 2 min with for adaptation^5^. The total duration of immobility was calculated by Noldus video-tracking system. An increase in immobility time revealed a depressive-like behavior.

**Morris water maze**

This test was performed to evaluate spatial learning and memory function in mice. As previously described^6^, the apparatus consisted of a circular water tank (100 cm in diameter and 35 cm in height), containing water (23 ± 1 °C) to a depth of 15.5 cm, which was rendered opaque by adding white food dye. A hidden platform (4.5 cm in diameter and 14.5 cm in height) was submerged 1 cm below the water surface and placed at the midpoint of one quadrant. Each mouse had four trials of training per day in the hidden platform test for 5 consecutive days. The latency to find the submerged escape platform was calculated for each trial. If the mouse did not reach the platform within 60 s, it was gently guided there and allowed to remain on the platform for 20 s. On day 6, a probe trial was carried out by removing the platform and allowing each mouse to swim freely for 60 s. The number of platform crossings, and the percent of time and distance spent in the target quadrant were recorded using a computerized video system (SMART, Barcelona, Spain).

**Ultra-performance liquid chromatography-mass spectrometry (UPLC-MS) analysis**

Upon thawing, the hippocampus, prefrontal cortex, cerebral cortex, and hypothalamus samples were added 600 µL methanol. The mixture was centrifuged for 10 min (12,000 × g, 4℃). The supernatant was dried in a vacuum centrifuge. The residue was re-dissolved in 50 µL acetonitrile/water (1:1, v/v) solvent. After centrifugation (12,000 × g, 4℃, 10 min), the supernatant was used for UPLC-MS analysis.

Analysis was conducted using an Acquity UPLC I-Class (Waters Corp. Milford, MA) coupled to a Waters Xevo G2-S QTof mass spectrometry system operating in negative ion mode as previously described with minor modifications^7^. Chromatographic separation was performed with a Welch Ultimate HILIC Amphion II column (2.1 mm × 100 mm, 3 µm, Welch Material Inc, USA). A solvent gradient program (flow rate of 400 µL/min) with 0.05 M ammonium formate in water (mobile phase A) and 100% acetonitrile (mobile phase B) was applied as follows: Initial–1.00 min: 5% A, 95% B; 1.01–5.00 min: 5%~50% A, 95%~50% B; 5.01–6.00 min: 50% A, 50% B; 6.01–7.20 min, 50%~5% A, 50%~95% B; 7.21–8.00: 5% A, 95% B. The injection volume was 5 µL and the temperature of the column was maintained at 30°C. The mass spectrometer was operated in negative ion mode with a 40 V cone voltage. Source and desolvation temperatures were 120 °C and 44℃ respectively. The raw UPLC-MS data were analyzed using QuanLynx applications manager version 4.1 (Waters) to obtain calibration equations and the quantitative concentration of each RIB in the samples.

**Metabolites extraction and widely targeted metabolomics analysis**

The hippocampus sample was placed into an EP tube with a 400 µL extraction solution (V *acetonitrile*: V *methanol*: V *water* = 2: 2: 1). After vortex, the mixture was homogenized at 30 Hz for 4 min and sonicated for 5 min in an ice-water bath. Next, the centrifugal process was conducted at 10000 rpm for 15 min at 4 ℃, and 300 µL of sample supernatant was transferred into a fresh EP tube. The rest of the sample was re-added with 400 µL of extraction solution, centrifuged, and 300 µL of supernatant was taken out. Twice supernatants were combined and completely dried in a vacuum concentrator (without heating). Subsequently, the dried samples were reconstituted in 200 µL of 50 % acetonitrile by sonication on ice for 10 min. The constitution was then centrifuged at 12000 rpm for 15 min at 4 ℃, and 75 µL of supernatant was transferred to a fresh glass vial for analysis. The quality control samples were prepared by mixing an equal aliquot of the supernatants from each sample.

After extraction, liquid chromatography-tandem mass spectrometry (LC-MS/MS) analysis were performed using an Agilent 1290 Infinity series ultra-high-performance liquid tandem chromatography (UHPLC) system (Agilent Technologies, Santa Clara, CA, USA) with a UPLC BEH Amide column (2.1 × 100 mm, 1.7 µm; Waters) at the Biotree Company (Shanghai, China). An Agilent 6495 triple quadrupole mass spectrometer (Agilent Technologies) was applied for assay development. Similar to the previously described^8^, for widely targeted metabolomics analysis, 200 MRM transitions representing the 200 metabolites were simultaneously monitored, which were selected based on their significant biological functions. After relative standard deviation de-noising, the missing values were filled up by half of the minimum value, and the total ion current normalization method was employed in the data analysis^9^. The final dataset containing the peak number, sample name and normalized peak area was imported to the SIMCA15.0.2 software package (Sartorius Stedim Data Analytics AB, Umea, Sweden) for multivariate analysis. After data was scaled and logarithmic transformed to minimize the impact of both noise and high variance of the variables, principal component analysis (PCA) and supervised orthogonal projections to latent structures-discriminant analysis (OPLS-DA) were used to visualize group separation and find significantly changed metabolites. Furthermore, the value of variable importance in the projection (VIP) of the first principal component in OPLS-DA analysis was obtained. The metabolites with VIP > 1.0 and *P* < 0.05 (Student's t-test) were considered as significantly differentially expressed metabolites (DEMs).

**RNA-Sequencing sample preparation, sequencing and data analysis**

Total RNA was extracted independently from the mice hippocampus using Trizol (Invitrogen, Carlsbad, CA, USA) according to the manufacturer’s instructions. RNA purity, concentration and integrity were evaluated with a Fragment Analyzer. The cDNA library construction and sequencing were performed by the Beijing Genomics Institute using BGISEQ-500 platform (BGI; Shenzhen, China)^10^. The raw data in this study can be available in NCBI SRA database with accession number of PRJNA639903.

Bowtie2 (version 2.2.5) was applied to align the clean reads to the reference coding gene set ^11^. Expression levels for each of the genes were normalized to fragments per kilobase of exon model per million mapped reads using RNA-seq by Expectation Maximization (version 1.2.8) with Ensembl database (Mus Musculus.GCF_000001635.26_GRCm38.p6)^12^. DESeq2 was applied to conduct differential gene expression analysis using read counts^13^. The threshold for significantly differentially expressed genes (DEGs) was set at as the adjusted *P*-value (Q value) < = 0.001 and |log2(fold change)| > = 1. Gene Ontology (GO) term enrichment analysis of annotated DEGs was performed by R based on the hypergeometric test, and *P*-value < 0.05 was set as the cutoff criterion.

**Western blotting**

For this analysis, the detailed process has been described previously^3^. In brief, the hippocampus was lysed using RIPA buffer with protease inhibitor cocktail (Beyotime, Shanghai, China), and protein was transformed from SDS-PAGE gel to a polyvinylidene fluoride membrane (Millipore, USA). After blocking, the membranes were incubated with anti-MAP kinase kinase 1 (MEK1; 1:2000; ab32091; Abcam, Cambridge, UK), anti-MEK2 (1:1000; ab32517; Abcam), anti-phospho-MEK1 (1:1000; ab96379; Abcam), anti-phospho-MEK2 (1:1000; ab30622; Abcam), anti-p38 mitogen activated protein kinase (p38MAPK; 1:1000; 8690S; Cell Signaling Technology (CST), Massachusetts, USA), anti-phospho-p38MAPK (1:1000; 4511S; CST), anti-cAMP responsive element binding protein (CREB; 1:1000; 9197S; CST), anti-phospho-CREB (1:1000; 9198S; CST), anti-calcium/calmodulin dependent protein kinase II α/β (CaMKIIα/β; 1:800; sc-5306; Santa Cruz Biotechnology, [California](javascript:;), USA), anti-phospho-CaMKIIα/β (1:1000; 12716S; CST) and anti-glyceraldehyde-3-phosphate dehydrogenase (GAPDH; 1:10000; ab8245; Abcam) overnight at 4℃, and then incubated with the corresponding secondary antibodies for 2 h at room temperature. Quantity One software (Bio-Rad, California, USA) was used to analyze the values.

**Supplementary References**

1. Wei, Y. et al. Ribosylation Triggering Alzheimer's Disease-Like Tau Hyperphosphorylation via Activation of CaMKII. *Aging Cell* **14**, 754-763 (2015).

2. Liu, M.Y. et al. Sucrose preference test for measurement of stress-induced anhedonia in mice. *Nat Protoc* **13**, 1686-1698 (2018).

3. Xu, K. et al. Validation of the targeted metabolomic pathway in the hippocampus and comparative analysis with the prefrontal cortex of social defeat model mice. *J Neurochem* **149**, 799-810 (2019).

4. Savignac, H.M. et al. Bifidobacteria exert strain-specific effects on stress-related behavior and physiology in BALB/c mice. *Neurogastroenterology & Motility* **26**, 1615-1627 (2014).

5. Doucet, M.V. et al. Small-molecule inhibitors at the PSD-95/nNOS interface have antidepressant-like properties in mice. *Neuropsychopharmacology* **38**, 1575-84 (2013).

6. Vorhees C. V, W.M.T. Morris water maze: procedures for assessing spatial and related forms of learning and memory. *Nat Protoc* **1**, 848-58 (2006).

7. Horgan, R.P. et al. Metabolic profiling uncovers a phenotypic signature of small for gestational age in early pregnancy. *J Proteome Res* **10**, 3660-73 (2011).

8. Cai, Y. et al. An integrated targeted metabolomic platform for high-throughput metabolite profiling and automated data processing. *Metabolomics* **11**, 1575-1586 (2015).

9. Dunn, W.B. et al. Procedures for large-scale metabolic profiling of serum and plasma using gas chromatography and liquid chromatography coupled to mass spectrometry. *Nat Protoc* **6**, 1060-83 (2011).

10. Leng, L. et al. Menin Deficiency Leads to Depressive-like Behaviors in Mice by Modulating Astrocyte-Mediated Neuroinflammation. *Neuron* **100**, 551-563 (2018).

11. Langmead B, S.S.L. Fast gapped-read alignment with Bowtie 2. *Nat Methods* **9**, 357-9 (2012).

12. Li, B. and Dewey, C.N. RSEM: accurate transcript quantification from RNA-Seq data with or without a reference genome. *BMC Bioinformatics* **12**, 323 (2011).

13. Love, M.I. et al. Moderated estimation of fold change and dispersion for RNA-seq data with DESeq2. *Genome Biology* **15**, (2014).
