# Supplementary material for: Chronic d-ribose and d-mannose overload induce depressive/anxiety-like behavior and spatial memory impairment in mice
Source: Transl Psychiatry. 2021 Feb 2;11:90. doi: 10.1038/s41398-020-01126-4 (PMC7854712; doi:10.1038/s41398-020-01126-4)
Supplement: Supplementary file 3 — Supplementary Figure Legends [file 41398_2020_1126_MOESM3_ESM.docx]

**Supplementary Figure Legends**

**Supplementary Fig. S1 Effects of different concentrations of D-ribose (RIB) and D-mannose (MAN) on mouse body weight, food consumption, anxiety-like behavior and spatial learning.** **(a, b)** Mouse body weight (a) and food intake (b) were measured weekly for 4 weeks in each group (0.4 g/kg RIB group: n = 15; 4 g/kg RIB group: n = 22; 0.48 g/kg MAN group: n = 14; 4.8 g/kg group: n = 21; control group: n = 22). **(c)** In the elevated plus-maze, the percentage of time spent in the open arms within 5 min was recorded (0.4 g/kg RIB group: n = 15; 4 g/kg RIB group: n = 22; 0.48 g/kg MAN group: n = 14; 4.8 g/kg group: n = 21; control group: n = 22). **(d)** In the Morris water maze, the length of time to find the hidden platform was recorded as escape latency for each of the five training days (0.4 g/kg RIB group: n = 15; 4 g/kg RIB group: n = 22; 0.48 g/kg MAN group: n = 14; 4.8 g/kg group: n = 21; control group: n = 22). **(e)** Levels of RIB in the cerebral cortex, prefrontal cortex, hippocampus, and hypothalamus from control, 0.4 g/kg RIB and 4 g/kg RIB groups (*n* = 4 each group). **(f)** Levels of MAN in the cerebral cortex, prefrontal cortex, hippocampus, and hypothalamus from control, 0.48 g/kg MAN and 4.8 g/kg MAN groups (*n* = 4 each group). Data represent mean ± S.E.M. * *P* < 0.05, ***P* < 0.01 versus the control group.

**Supplementary Fig. S2 Widely targeted metabolomics and transcriptomics analysis of hippocampus from 4 g/kg D-ribose (RIB), 4.8 g/kg D-mannose (MAN) and control (CON) groups mice. (a, b)** Principal component analysis (PCA; a) and orthogonal partial least-squares discriminant analysis (OPLS-DA; b) scores plot of RIB (blue circles) versus CON (red circles) and MAN (purple circles) versus CON (red circles) groups (*n* = 9 each group). **(c)** Statistical validation of the OPLS-DA model by permutation testing. **(d)** Levels of glycine in the hippocampus from control, 0.4 g/kg RIB, 4 g/kg RIB, 0.48 g/kg MAN, and 4.8 g/kg MAN groups (*n* = 4 each group). **(e)** Levels of thiamine in the hippocampus from control, 0.4 g/kg RIB and 4 g/kg RIB groups (*n* = 4 each group). **(f-h)** Levels of creatinine (f), acetylcholine (g), and nicotinamide (h) in the hippocampus from control, 0.48 g/kg MAN and 4.8 g/kg MAN groups (*n* = 4 each group). **(i, j)** Volcano plot graph of the 96 up-regulated (red dots) and 94 down-regulated (blue dots) diﬀerentially expressed genes (DEGs) between the RIB and CON groups (i) and the 113 up-regulated (red dots) and 151 down-regulated (blue dots) DEGs between the MAN and CON groups (j) (*n* = 4 each group). **(k)** mRNA expression levels of Fezf1, Ecel1, Calcr, Foxb1, Igfn1, Meltf, Gm46290, Trh, Tnfrsf4, and Hist2h2aa2 in the hippocampus from control, 0.4 g/kg RIB and 4 g/kg RIB groups (*n* = 3 each group). **(l)** mRNA expression levels of Pax6os1, Dio3, Ttr, Irs4, Kifc1, Ucn3, Zfp973, Tfap2b, Gata3, and Pax3 in the hippocampus from control, 0.4 g/kg RIB and 4 g/kg RIB groups (*n* = 3 each group). **(m)** mRNA expression levels of Adam33, Ecel1, Rspo1, Igfn1, Pmch, Gm46290, Kifc1, Zfp973, Grxcr2, and Slc4a5 in the hippocampus from control, 0.48 g/kg MAN and 4.8 g/kg MAN groups (*n* = 3 each group). **(n)** mRNA expression levels of Rasgef1c, Mup5, Smg8, Psmb8, Trh, Ucn3, Tfap2d, Dio3, Cdh26, and Slc6a4 in the hippocampus from control, 0.48 g/kg MAN and 4.8 g/kg MAN groups (*n* = 3 each group). **(o, p)**Top 20 gene ontology (GO) biological processes analysis of the DEGs from the RIB versus CON (o; *P* < 0.05) and the MAN versus CON (p; *P* < 0.05) comparison. Data represent mean ± S.E.M. * *P* < 0.05, ***P* < 0.01, *** *P* < 0.001 versus the control group. Abbreviations: *Adam33* ADAM metallopeptidase domain 33, *Aldh1a2* aldehyde dehydrogenase 1 family member a2, *Calcr* calcitonin receptor, *Cdh26* cadherin-like 26, *Ecel1* endothelin converting enzyme like 1, *Fezf1* FEZ family zinc finger 1, *Foxb1* forkhead box B1, *Gata3* GATA binding protein 3, *Gm46290* predicted gene, 46290, *Grxcr2* glutaredoxin, cysteine rich 2, *Igfn1* immunoglobulin-like and fibronectin type III domain containing 1, *Irs4* insulin receptor substrate 4, *Kifc1* kinesin family member C1, *Meltf* melanotransferrin, *Mup5* major urinary protein 5, *Pax3* paired box 3, Pmch pro-melanin-concentrating hormone, *Pomc* proopiomelanocortin, *Psmb8* proteasome subunit, beta type 8, *Rasgef1c* RasGEF domain family, member 1C, *Rspo1* R-spondin 1, *Slc6a4* solute carrier family 6 (neurotransmitter transporter, serotonin), member 4, *Slc4a5* solute carrier family 4, sodium bicarbonate cotransporter, member 5, *Smg8* smg-8 homolog, nonsense mediated mRNA decay factor, *Tfap2b* transcription factor AP-2 beta, *Tfap2d* transcription factor AP-2 delta, *Trh* thyrotropin releasing hormone, *Ucn3* urocortin 3, *Zfp973* zinc finger protein 973.

**Supplementary Fig. S3 Integrated analysis of widely targeted metabolomics and transcriptomics data.** **(a, b)** Nine quadrant diagrams of the selected differentially expressed metabolites (DEMs) and corresponding diﬀerentially expressed genes (DEGs) from the D-ribose (RIB) versus control (CON) (a) and the D-mannose (MAN) versus CON (b) comparison. The rows indicate the log2(fold change) value of each DEG, and the columns indicate the log2(fold change) value of each DEM. **(c, d)** Heatmap of the selected DEMs with the DEGs from the RIB versus CON (c) and the MAN versus CON (d) comparison. The rows indicate the metabolites, and the columns indicate the genes.

**Supplementary Fig. S4 Top 20** **gene ontology (GO) analysis of the selected diﬀerentially expressed genes (DEGs) from the D-ribose (RIB) versus control (CON) and the D-mannose (MAN) versus CON comparisons that were highly correlated with the specific differentially expressed metabolites (Pearson correlation coefficient |r|** **> 0.8 and *P* < 0.05).** **(a–c)** Cellular components (a), molecular functions (b), and biological processes (c) analysis of the 51 DEGs from the RIB versus CON comparison. Only the top 20 GO terms that were significantly overrepresented (*P* < 0.05) are shown. **(d–f)** Cellular components (d), molecular functions (e), and biological processes (f) analysis of the 109 DEGs from the MAN versus CON comparison. Only the top 20 GO terms that were significantly overrepresented (*P* < 0.05) are shown.

**Supplementary Fig. S5** **Analysis of the relationship between selected diﬀerentially expressed genes (DEGs). (a, b)** Protein-protein interaction (PPI; score > 500) and co-expression (Pearson correlation coefficient |r| > 0.8) network of the selected DEGs from the RIB versus CON (a) and the MAN versus CON (b) comparisons.
