# Supplementary material for: Chronic d-ribose and d-mannose overload induce depressive/anxiety-like behavior and spatial memory impairment in mice
Source: Transl Psychiatry. 2021 Feb 2;11:90. doi: 10.1038/s41398-020-01126-4 (PMC7854712; doi:10.1038/s41398-020-01126-4)
Supplement: Supplementary file 4 — Supplementary Table S1 [file 41398_2020_1126_MOESM4_ESM.docx]

**Table S1 qRT-PCR primer sequences used in this study.**

| Genes | | Forward primer (From 5’ to 3’) | Reverse primer (From 5’ to 3’) |
| --- | --- | --- | --- |
| Fezf1  Ecel1  Calcr  Foxb1  Igfn1  Meltf  Gm46290  Trh  Tnfrsf4  Hist2h2aa2  Pax6os1  Dio3  Ttr  Irs4  Kifc1  Ucn3  Zfp973  Tfap2b  Gata3  Pax3  Adam33  Rspo1  Pmch  Grxcr2  Slc4a5  Rasgef1c  Mup5  Smg8  Psmb8  Tfap2d  Cdh26  Slc6a4  Tcf7l2  Pomc  Aldh1a2  Grin2a Grin2b  Camk2a Camk2b  Gapdh | AGAACTCTGCGATGGGCTGG  TGAGGCCGGGATTTGCTCC  GCAGGCACTGCTAAGGAGA  GACCGGCAACTTGGAACAAC  TAGCAACCCGGGCTATCGTA  AGGCATTAACCGGACTGTGG  AGCCTATTCCGAAGACCCCT  AAACCCCTCCCTTACCCACT  GGCCCTGCATTTGCTGTTCT  TGAGCCAATGAGAGCGTCG  GTGATCACCTTGGGTCCTGG  TCAGACGACAACCGTCTGTG  TCCCCTGCTCCTAAAACCCA  CTGGAGCCCTTAACCACAGG  ATTCAAAAGTGGCGGGTGTG  CCAAACGCACCTCCAGATCA  GCGGTTCTTCCGGGTCAA  GCAGAAGAAAAGAGGCCAAAAA  CAGGCAGGGAGTGTGTGAAC  GCCTCAGACCGACTATGCTC  GGAGAGCTAGTCACTCCCCA  GAGACAGAGGCGGATCAGTG  CCAATGCACTCTTGTTTGGC  AGCGTATTCAGAGACAGCGG  CCGCCCCCTTTCTTTCTCAT  CCCCAATCCGCCCATACACA  TGAGCCTCCAGTGTTGAGTG  GGAGGATGAGATCTGCGTGG  CAGAGTCGACGGATCTTCGG  CTCCTCCTAGGGAGAGACAGG  GTGCTCCTGCACATTTGGAC  GCCATCAGCCCTCTGTTTCT  CAGGGAAGAACAGGCAAAAT GAGCTGGTGCCTG GAGAG  GACTTGTAGCA GCTGTCTTCACT TCTTGAACTACAAGGCCGGG  CCTCCTGTGTGAGAGGAAAGA CCCCAAAGTCACAGAGCCAT  TGGAATCTTCCGACAGCACC  ATCACTGCCACCCAGAAGAC | GTCACCTCGGTTCAGGTAGC  GTAGGTGAGCTTGTCGTCGG  GGTGTTCTCAGGAACGCAGA  GCGGTCAGCGAGATGTATGA  CAGCCTTCTGGGATCTCGTC  AATCACCAACGGCTTTGAGC  CCAAGGTCCCAGTTTTTGCG  GAAGAACCGTCTTGGCCAGT  AGAACGGTGTGGAGGGTACT  CTTGTTGAGCTCCTCGTCGT  CAGCCCTGCCCTTTACATCA  AAAATTGAGCACCAACGGGC  CCATGACCTCCCCAAGATGG  TCCTGTAGTCTCGTCTGGCA  CACAGAAAGGGTGCAAAGGC  GGGTACAGCTTCCCCTTGTG  AACACCCAGTAGAGTCACGC  CACAGGAGCTAGGCTTGAGA  CGGGTCTGGATGCCTTCTTT  TGAAAGGCACTTTGTCCATACT  ATCCTGGCCTTCAGCCTCTA  CGGATGTCGTTCCTCTCCAG  ATTGTTCCAGAGAAGGGGCG  CCATCGGGGTTCGGATGATT  CTCATCTTCCTCCCCCAGGA  TCCACTCGGCTAACAACTGG  CCCATGCTGTATGGAAGGGA  TGGCTTCCGGGGACTATGTA  AAAGGACCTCAGGAATGCGG  CGTGGAATTGGCTACGGACT  CGATCCAGATAGACCTGGGC  TGGCTTAGAGGGGAGGAGTC  GGGGGAGGCGAGTCTAGTAA TTTTCATCAGGGGCTGTTC  TCACCCATTTCTCTCCCATTTCC GCGAGGTCAATCTGCCTCTT  CCCGGGGAACTACTGAGAGA ACGTGTCGTCTTCCACTGTC  TGAGCTGCTC TGTGGTCTTG ACACATTGGGGGTAGGAACA |  |

**Abbreviations:** *Adam33* ADAM metallopeptidase domain 33, *Aldh1a2* aldehyde dehydrogenase 1 family member a2, *Camk2a/b* calcium/calmodulin dependent protein kinase 2a/b, *Calcr* calcitonin receptor, *Cdh26* cadherin-like 26, *Ecel1* endothelin converting enzyme like 1, *Fezf1* FEZ family zinc finger 1, *Foxb1* forkhead box B1, *Gapdh* glyceraldehyde-3-phosphate dehydrogenase, *Gata3* GATA binding protein 3, *Gm46290* predicted gene, 46290, *Grin2a/b* glutamate ionotropic receptor NMDA type subunit 2a/b, *Grxcr2* glutaredoxin, cysteine rich 2, *Igfn1* immunoglobulin-like and fibronectin type III domain containing 1, *Irs4* insulin receptor substrate 4, *Kifc1* kinesin family member C1, *Meltf* melanotransferrin, *Mup5* major urinary protein 5, *Pax3* paired box 3, Pmch pro-melanin-concentrating hormone, *Pomc* proopiomelanocortin, *Psmb8* proteasome subunit, beta type 8, *Rasgef1c* RasGEF domain family, member 1C, *Rspo1* R-spondin 1, *Slc6a4* solute carrier family 6 (neurotransmitter transporter, serotonin), member 4, *Slc4a5* solute carrier family 4, sodium bicarbonate cotransporter, member 5, *Smg8* smg-8 homolog, nonsense mediated mRNA decay factor, *Tcf7l2* transcription factor 7 like 2, *Tfap2b* transcription factor AP-2 beta, *Tfap2d* transcription factor AP-2 delta, *Trh* thyrotropin releasing hormone, *Ucn3* urocortin 3, *Zfp973* zinc finger protein 973.
