# Supplementary figures and images for: Chronic d-ribose and d-mannose overload induce depressive/anxiety-like behavior and spatial memory impairment in mice
Source: Transl Psychiatry. 2021 Feb 2;11:90. doi: 10.1038/s41398-020-01126-4 (PMC7854712; doi:10.1038/s41398-020-01126-4)

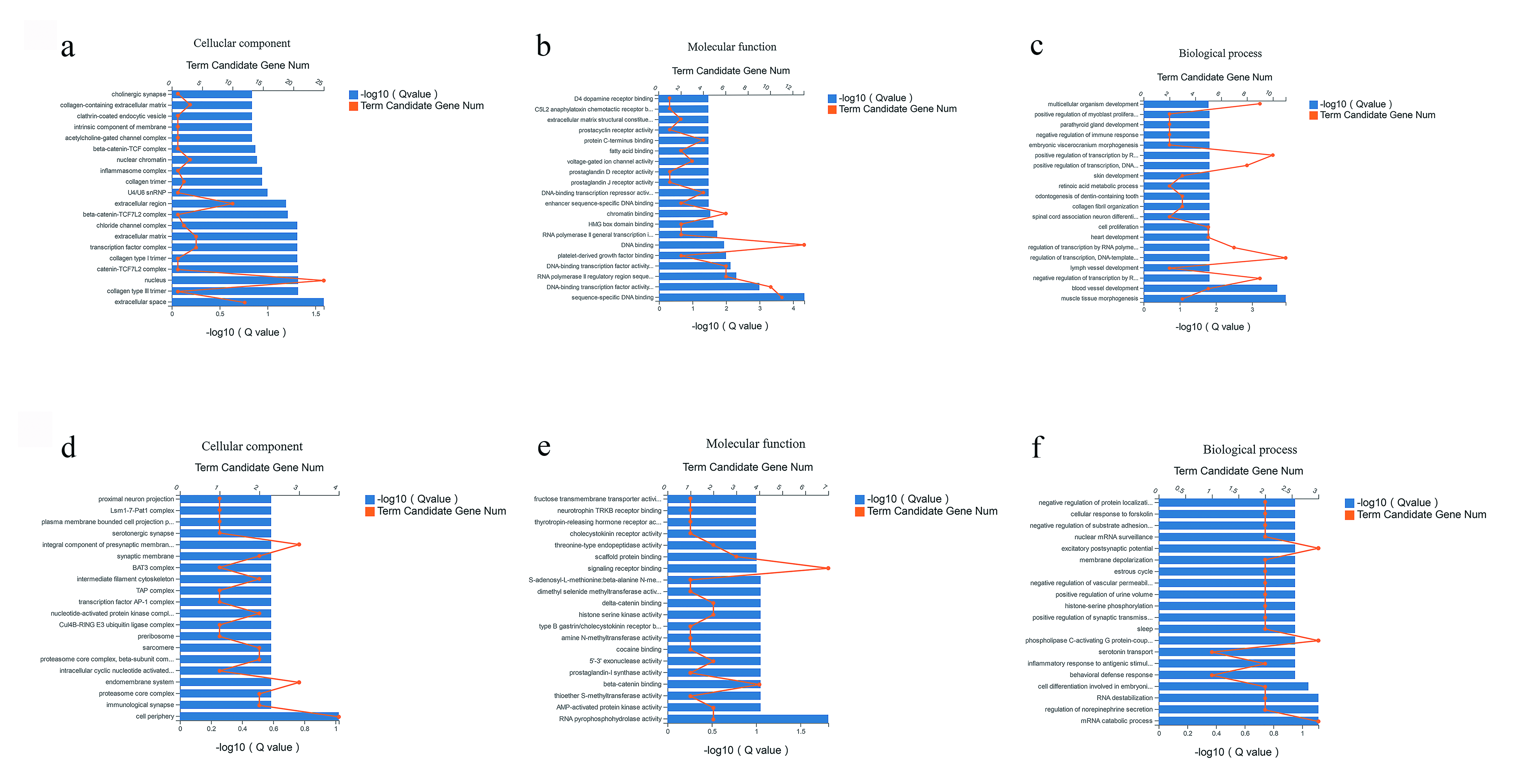

Supplement: Supplementary file 17 — Supplementary Figure S4 [file 41398_2020_1126_MOESM17_ESM.tif]

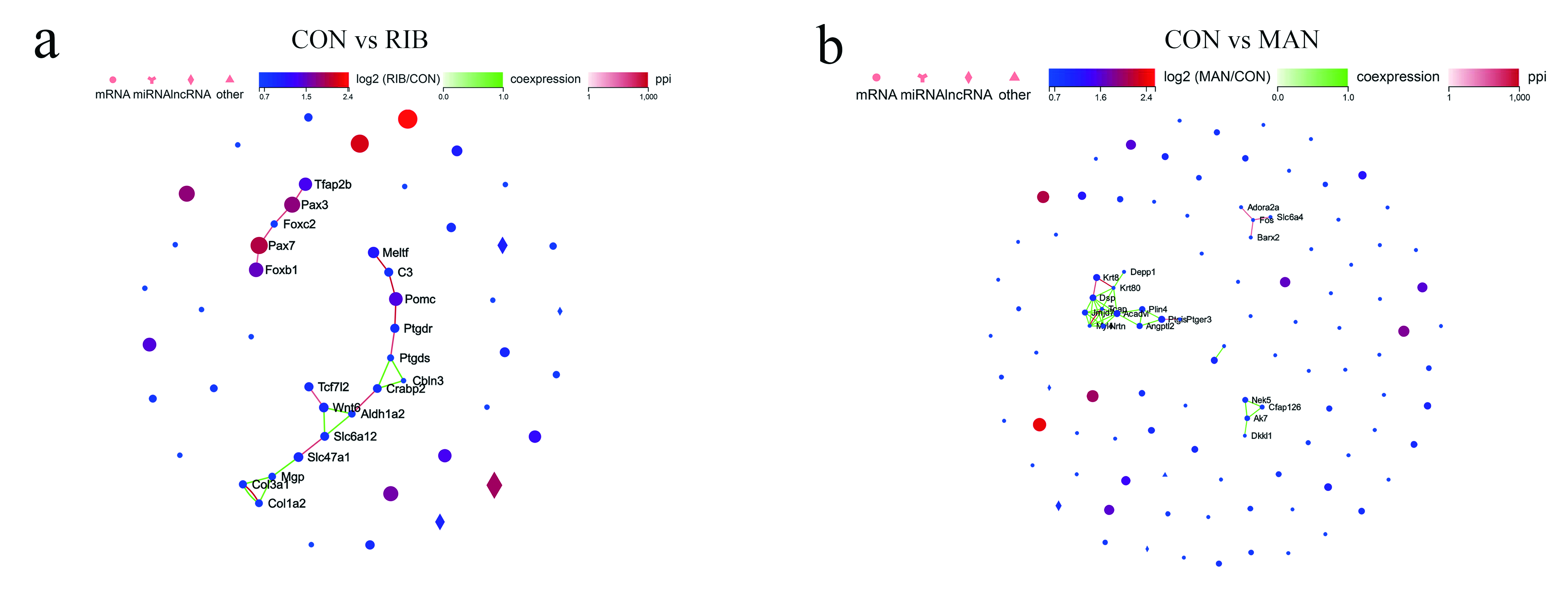

Supplement: Supplementary file 18 — Supplementary Figure S5 [file 41398_2020_1126_MOESM18_ESM.tif]
